# A new species of *Papiliomyces* (Clavicipiteae, Hypocreales) from China

**DOI:** 10.3897/BDJ.11.e86868

**Published:** 2023-06-06

**Authors:** Yan Zhang, TingChi Wen, Yuanpin Xiao, Yu Yang, Xingcan Peng

**Affiliations:** 1 College of Pharmacy, Guizhou University, Guiyang, China College of Pharmacy, Guizhou University Guiyang China; 2 Engineering Research Center of Southwest Bio-Pharmaceutical Resources, Ministry of Education, Guizhou University, Guiyang, China Engineering Research Center of Southwest Bio-Pharmaceutical Resources, Ministry of Education, Guizhou University Guiyang China; 3 The Mushroom Research Centre, Guizhou University, Guiyang, China The Mushroom Research Centre, Guizhou University Guiyang China; 4 Center of Excellence in Fungal Research, and School of Science, Mae Fah Luang University, Chiang Rai, Thailand Center of Excellence in Fungal Research, and School of Science, Mae Fah Luang University Chiang Rai Thailand

**Keywords:** *Papiliomyces*, molecular phylogeny, one new species, taxonomy

## Abstract

**Background:**

*Papiliomyces* (Hypocreales, Sordariomycetes) was introduced to accommodate two species: *Papiliomycesliangshanensis* and *Papiliomycesshibinensis*. Later, *Papiliomycesliangshanensis* was renamed *Ophiocordycepsliangshanensis*. However, the *Papiliomycesliangshanensis* molecular data (Nepalese) used to establish the *Papiliomyces* genus was different from *Ophiocordycepsliangshanensis* (China) strains.

**New information:**

This paper describes a new species, *Papiliomyceslongiclavatus*, found in Yangchang District, Guiyang City, Guizhou Province, China. It is proposed, based on morphology and multilocus phylogeny (ITS, SSU, LSU, *TEF1*, *RPB1* and *RPB2*). The new species is phylogenetically most closely related to *Papiliomycesliangshanensis* (Nepalese collections). However, *Papiliomycesliangshanensis* (Nepalese collections) requires morphological details and additional detection. The new species differs from other *Papiliomyces* species in having robust stroma with completely immersed perithecia, multi-septate ascospores, cylindrical secondary ascospores, two types of phialides and two types of conidia:longer α-conidia and longer β-conidia.

## Introduction

Fungi are amongst the most important organisms, influencing human activities and playing a crucial role in ecosystems (Mueller and Schmit 2007). About 2000 species of fungi are being described annually (Cheek et al. 2020). Entomopathogenic fungi have received great attention due to their ecological, economic and medical applications (Yang et al. 2021). However, the diversity of entomopathogenic fungi necessitates additional research.

Sung et al. (2007a) introduced the genus *Metacordyceps* to accommodate some species of *Cordyceps*, characterised by solitary to multiple stromata, simple to branched, with a fleshy or tough whitish stipe, strongly pigmented green to yellow cylindrical to enlarged fertile part and superficial perithecia partially or completely immersed in stroma (Sung et al. 2007a, Kepler et al. 2012a). Based on the generated DNA sequences and multi-gene phylogenetic evidence, Sung et al. (2007a) reclassified some species from *Metacordyceps* to *Papiliomyces*, such as *Metacordycepsliangshanensis* and *Metacordycepsshibinensis*. This was utilised in the Outline of Ascomycota (Wijayawardene et al. 2018).

The genus *Papiliomyces* was proposed by Mongkolsamrit et al. (2020) in *Clavicipiteae* family (Hypocreales, Sordariomycetes), based on phylogenetic analyses to accommodate two species: *Papiliomycesliangshanensis* (Nepalese collections) and *Papiliomycesshibinensis* (China). However, based on morphology and phylogenetic analyses, the type species, *Papiliomycesliangshanensis*, was recombined into *Ophiocordyceps* (Wang et al. 2021). The genus name originated from the Latin word 'papilio', meaning butterfly or moth (Mongkolsamrit et al. 2020). The primary characteristics of the teleomorph of *Papiliomyces* are solitary to multiple (branched and robust to wiry stromata), superficial perithecia to completely immersed in the stroma, ampulliform, ellipsoid to ovoid, asci cylindrical and ascospores whole with septation or breaking into cylindrical part-spores (Kepler et al. 2012a, Kepler et al. 2012b, Mongkolsamrit et al. 2020, Wang et al. 2021).

Through culturing and molecular approaches, *Metarhizium* has been linked to *Metacordyceps* as sexual states (Kepler et al. 2014). However, they are forming distinct branches in the phylogenetic tree. Moreover, *Metacordyceps* is different from *Metarhizium* in producing superficial perithecia and a wiry, branched stromata or a white to faint yellow robust stroma (Zang et al. 1982, Sung et al. 2007b, Wen et al. 2015, Mongkolsamrit et al. 2020). Sexual morphs of *Metarhizium* produce semi-immersed to completely immersed perithecia on a robust and almost fleshy dark green or purplish stroma (Sorokin 1883, Sung et al. 2007a, Kepler et al. 2012a). Phylogenetically, the genus is closely related to *Purpureomyces* and *Keithomyces* (Mongkolsamrit et al. 2020). The three species of *Purpureomyces* (Hywel-Jones 1994, Kepler et al. 2012a, Kepler et al. 2014) differ from *Papiliomyces* spp. in that they can produce fleshy, purple stromata and the ascomata are obliquely embedded in the stroma (Mongkolsamrit et al. 2020). *Keithomyces* includes three species primarily isolated from soil, producing *Paecilomyces*-like asexual morphs, conidiophores with divergent whorls of two to four phialides and echinulate to aciculate conidia (Mongkolsamrit et al. 2020).

In this study, a new species, *Papiliomyceslongiclavatus* is introduced, based on the morphology and phylogenetic analysis of a six-locus dataset.

## Materials and methods

### Sample collection and morphological characteristic examination

The specimens were collected from Yangchang Town, Wudang District, Guiyang City, Guizhou Province, China on 14 June 2020. They were stored in plastic containers at low temperature and transported to the laboratory for identification. The macro-morphological characteristics were described, based on fresh material and on the photographs provided here. Fresh specimens were used to isolate the fungus through the tissue culture method in a potato dextrose agar (PDA) medium. Herbarium materials were deposited at Guizhou University (GACP) and Kunming Institute of Botany, Chinese Academy of Sciences (HKAS). For micro-morphological examination, fruiting bodies and living culture mycelia were examined using a stereo dissecting microscope (Leica S9E). Hand sections of fruiting structures were mounted in water for microscopic study and photomicrography. The microcharacteristics of the fungi were examined under a Leica DM2500 compound microscope and photographed. Facesoffungi and Index Fungorum numbers were provided as explained by Jayasiri et al. (2015) and Index Fungorum (http://indexfungorum.org, accessed 6 April 2023).

### DNA extraction, PCR amplification and sequencing

Dried specimens were used to extract genomic DNA using the EZgene TM Fungal gDNA Kit (Biomiga, CA, USA) according to the manufacturer’s instructions. The extracted DNA was stored at -20°C. Reaction mixtures (25 μl) contained 2 μl template DNA (ca. 10 ng), 11 μl distilled water, 1 μl (10 μM) of each primer and 10 μl 2x Taq PCR StarMix with Loading Dye (GenStar). The primers for amplifying and sequencing were ITS5 and ITS4 for the internal transcribed spacer gene region (ITS) (White et al. 1990), LR0R and LR5 for the partial large subunit rDNA gene region (LSU) (Vilgalys and Hester 1990, Hopple 1994), NS1 and NS4 for the ribosomal small subunit rDNA gene region (SSU) (White et al. 1990), EF1-983F and EF1-2218R for the partial translation elongation factor 1-alpha gene region (*TEF1*) (Sung et al. 2007b), CRPB1 and RPB1Cr for the partial RNA polymerase II largest subunit gene region (*RPB1*) (Castlebury et al. 2004) and RPB2-5F2 and RPB2-7Cr for the partial RNA polymerase II second largest subunit gene region (*RPB2*) (Liu et al. 1999, O'Donnell et al. 2007). The PCR amplification conditions were the following: for ITS, initial denaturation at 94°C for 3 min, followed by 33 cycles of 94°C for 30 s, 51°C for 50 s and 72°C for 45 s and final extension of 72°C for 10 min; for LSU and SSU, initial denaturation at 94°C for 3 min, followed by 33 cycles at 94°C for 30 s, 51°C for 30 s and 72°C for 2 min and final extension of 72°C for 10 min; for *TEF1*, initial denaturation at 94°C for 3 min, followed by 33 cycles of 94°C for 30 s, 58°C for 50 s and 72°C for 1 min and final extension of 72°C for 10 min; for *RPB1*, initial denaturation at 94°C for 3 min, followed by 33 cycles of 94°C for 1 min, 52°C for 1 min and 72°C for 1 min and final extension at 72°C of 10 min; and for *RPB2*, initial denaturation at 94°C for 3 min, followed by 33 cycles of 94°C for 30 s, 54°C for 40 s and 72°C for 80 s and final extension of 72°C for 10 min. The amplified PCR products were verified by 1% agarose gel electrophoresis stained with ethidium bromide in 1x TBE. They were directly sequenced with the above-mentioned primers by Sangon Biotech (Shanghai) Co., Ltd., Shanghai, China.

### Sequence alignment and phylogenetic analyses

Reference sequences were downloaded from NCBI GenBank, https://www.ncbi.nlm.nih.gov/genbank/ (Table 1). BioEdit (Alzohairy 2011) was used to assemble downloaded and newly-generated sequences. Sequences were aligned by locus with MAFFT version 7 (Katoh and Standley 2013) (https://mafft.cbrc.jp/alignment/server/) and trimmed using TrimAl version 1.3 (Capella-Gutiérrez et al. 2009). FasParser was used to splice the ITS, SSU, LSU, *TEF1*, *RPB1* and *RPB2* sequences (Katoh and Standley 2013). Gaps were considered as missing data.

Maximum Likelihood (ML) analyses were performed using IQ-TREE 2 (Minh et al. 2020) under partitioned models. The built-in ModelFinder (Kalyaanamoorthy et al. 2017) was used to select appropriate models for each of the six loci. Branch support was estimated using 1000 ultrafast bootstrap (UFBoot2) replicates (Hoang et al. 2018). For Bayesian analysis (BI), MrBayes version 3.2.7 (Ronquist et al. 2012) was utilised to evaluate posterior probabilities (PP) using Markov Chain Monte Carlo sampling (MCMC). MrMTgui (Nuin 2007), combined with MrModelTest and Paup, was utilised to determine the best-fit evolution model for each locus using the Akaike Information Criterion (AIC). BI was conducted with six simultaneous MCMC chains, and trees were sampled every 100^th^ generation. The analyses were stopped after 5,000,000 generations when the average standard deviations of the split frequencies were below 0.01. The first 25% of the resulting trees were discarded as burn-in and PP was calculated from the remaining sampled trees. The phylogenetic tree was visualised using FigTree version 1.4.0 (http://tree.bio.ed.ac.uk/software/figtree/).

## Taxon treatments

### Papiliomyces longiclavatus

Y. Zhang, Y.P. Xiao & T.C. Wen sp. nov.

A8FB36D4-5C6C-5235-A3E1-9C170F2641D3

IF558796

#### Materials

- **Type status:** Holotype. **Occurrence:** catalogNumber: GACP YC20061403; recordedBy: Yan Zhang; lifeStage: sexualMorph; occurrenceID: 94F0EF70-F2D9-5663-BB82-44C056D5B97A; ex-type: GZUCC-1403; **Taxon:** scientificName: *Papiliomyceslongiclavatus*; **Location:** country: China; stateProvince: Guizhou; county: Guiyang City; locality: Yangchang Town; verbatimElevation: 1303 m; verbatimCoordinates: 26°50′12.23″N, 106°53′41.58″E; decimalLatitude: 0.836731; decimalLongitude: 106.894883; georeferenceProtocol: label; **Identification:** identifiedBy: Yuan-pin Xiao; dateIdentified: 2020; **Event:** eventDate: 14 June 2020
- **Type status:** Paratype. **Occurrence:** catalogNumber: HKAS 115914; recordedBy: Yan Zhang; lifeStage: sexualMorph; occurrenceID: BA6BD204-6AB6-58CF-9EA3-850CB1357663; **Taxon:** scientificName: *Papiliomyceslongiclavatus*; **Location:** country: China; stateProvince: Guizhou; county: Guiyang City; locality: Yangchang Town; verbatimElevation: 1325.7 m; verbatimCoordinates: 26°50′12.03″N, 106°53′41.70″E; decimalLatitude: 0.836675; decimalLongitude: 106.894917; georeferenceProtocol: label; **Identification:** identifiedBy: Yuanpin Xiao; dateIdentified: 2020; **Event:** eventDate: 14 June 2020

#### Description

Facesoffungi number: FoF 10474

**Figure 1.**
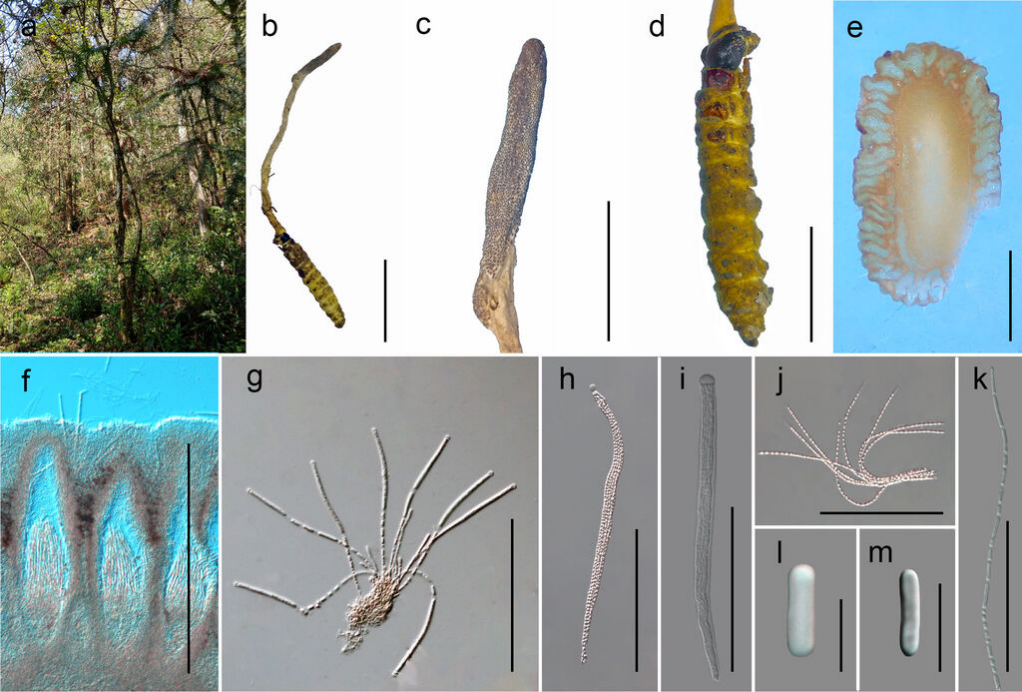
*Papiliomyceslongiclavatus* (GACP YC20064103), sexual morph. **a** Habitat; **b** Overview of the host and stroma; **c** Stroma; **d** Host; **e, f** Vertical section of the stroma; **g, h** Immature to mature asci; **i** Apical cap; **j, k** Part of ascospores; **l, m** Secondary spores. Scale bars: b = 3 cm, c = 1 cm, d = 1 cm, e = 0.1 cm, f = 400 μm, g = 150 μm, h–j = 100 μm, k = 50 μm, l, m = 5 μm.

**Sexual morph** (Fig. 1): Host: a bat moth larva (Lepidoptera, Hepialidae), 3.5–4.8 cm long, greyish to light yellow. Stromata: 4–6 cm long, 0.3–0.5 cm wide, arising from the head of host, clavate, solitary. Stipe: cylindrical, greyish to light yellow, fleshy, glabrous, enlarging abruptly at fertile portion. Fertile head: 1.5–2.1 cm long, 0.4–0.6 cm wide, grey white to dark grey, different from the stipe, without sterile tip. Ascomata: 320–580 × 110–230 μm (442 × 186 μm, n = 30), flask-shaped, immersed. Asci: 140–230 × 5–7 μm (183 × 6 μm, n = 30), narrowly cylindrical, 8-spored, hyaline, with a thick apical cap. Apical cap: 5–7 μm (6 μm, n = 30). Ascospores as long as asci, hyaline, filiform, smooth, irregularly breaking into secondary spores. Secondary spores: 2–9 × 1–2 μm (5.5 × 1.5 μm, n = 30), cylindrical, hyaline, irregular length.

**Figure 2.**
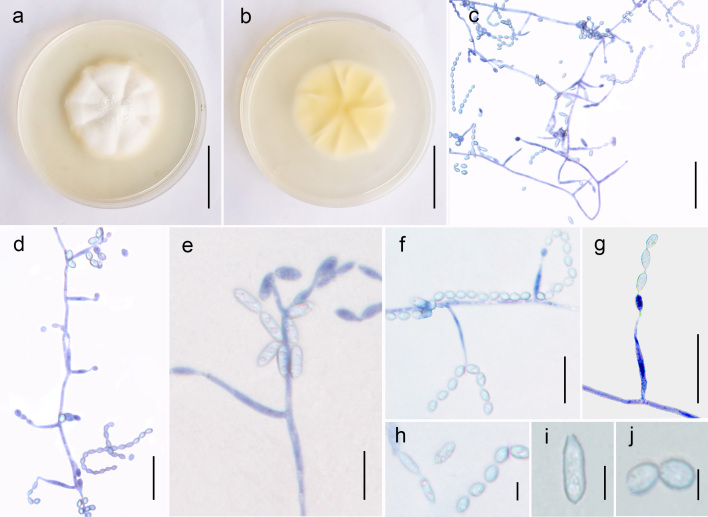
*Papiliomyceslongiclavatus* (ex-type: GZUCC-4103), asexual morph. **a** Upper side of the culture; **b** Reverse of the culture; **c** Mycelium with phialides and conidia; **d, f** α-phialides; **e, g** β-phialides; **h** Two types of conidia; **i** β-conidia; **j** α-conidia. Scale bars: a, b = 1 cm, c = 30 μm, d–g = 20 μm, h–j = 5 μm.

**Asexual morph** (Fig. 2): Colonies on Czapek agar: attaining a diameter of 2–3 cm within 14 d at 25°C, dense, flat, velvety, white. α-phialides: 13–24 × 1–2 μm (18 × 1.5 μm, n = 30), hyaline, *Hirsutella*-like smooth, solitary, mostly arising from aerial hyphae, slender, with a short neck. β-phalides: 28–45 × 1–2 μm (36 × 1.5 μm, n = 30), hyaline, smooth, slender, *Acremonium*-like, mostly solitary. α-conidia: 3–5 × 1–3 μm (4.5 μm, n = 30), subglobose, one-celled, smooth, hyaline. β-conidia: 6–10 × 1–3 μm (8 × 2 μm, n = 30), fusiform, with both ends sharp, one-celled, smooth.

**Host**: On larvae of a bat moth (Lepidoptera, Hepialidae) living in soil.

#### Etymology

Referring to the shape of the stroma.

#### Distribution

Thus far only known from China.

## Analysis

**Phylogeny**

Ten taxa (two with new sequence data) were included in the combined *ITS*, *SSU*, *TEF-1α*, *RPB1*, *LSU* and *RPB2* dataset (Table 1), which included 5016 characters with gaps: 561 characters for *ITS*, 887 for *LSU*, 1040 for *SSU*, 655 for *RPB1*, 957 for *RPB2* and 916 for *TEF-1α*. The IQ-TREE analysis had the same topology as the Bayesian analysis. The ML tree with the highest likelihood value, -9176.099, is presented. The matrix contained 280 unique alignment patterns, including 289 parsimony-informative, 133 singleton sites and 4594 constant sites. For the IQ-Tree, the respective partition best-fit models were: JC+I for *SSU* and *RPB2*, TNe for *LSU*, K2P for *ITS*, TN+F for *TEF-1α* and K2P+I for *RPB1*. In the Bayesian analysis, the respective partition best-fit models were: F81+I for *SSU*, GTR for *LSU* and *TEF-1α*, K80+G for *ITS* and *RPB1* and SYM for *RPB2*.

## Discussion

**Figure 3.**
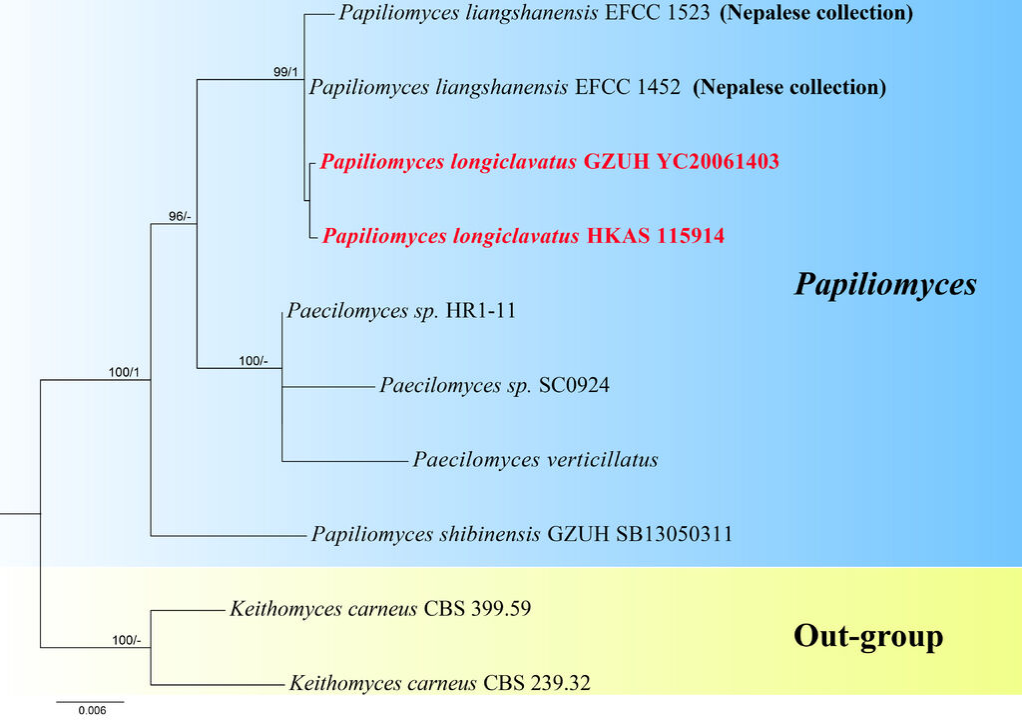
Phylogram of *Papiliomyceslongiclavatus* generated from the Maximum Likelihood (IQ-tree) analysis of combined *ITS*, *SSU*, *TEF-1α*, *RPB1*, *LSU* and *RPB2* sequence data. The tree was rooted to *Keithomycescarneus* (CBS 399.59) and *Keithomycescarneus* (CBS 239.32). Maximum Likelihood bootstrap values greater than 75% and posterior probabilities from Bayesian Inference more than 0.95 were indicated above the nodes. The new species is indicated in red.

Using morphological and phylogenetic analyses, we propose *Papiliomyceslongiclavatus* sp. nov. from China. It shares a close relationship with *Papiliomycesliangshanensis* (EFCC 1452, EFCC 1523, Sung et al. (2007), Mongkolsamrit et al. (2020)), *Papiliomycesshibinensis* (Wen et al. 2015) and *Paecilomycesverticillatus* (Li et al. 2006). *Papiliomycesliangshanensis* was reclassified as *Ophiocordycepsliangshanensis* after morphological and phylogenetic analyses (Wang et al. 2021). However, the Nepalese collections (EFCC 1452, EFCC 1523) are lacking a morphological description (Sung et al. 2007) and which are named *Papiliomycesliangshanensis*. These two collection groups place our new species within the same clade (Fig. 3).

Phylogenetically, they are different from *Papiliomycesliangshanensis* (EFCC 1452, EFCC 1523): 0 bp in ITS, 0 bp in LSU, 4 bp/762 bp (99%) in TEF and 7 bp/762 bp (99%) in RPB2. However, the Nepalese collections (EFCC 1452, EFCC 1523) lack morphological descriptions (Sung et al. 2007) and which was then named *Papiliomycesliangshanensis*. Consequently, we indicated these two collections on the phylogenetic tree. These two collections, EFCC 1452 and EFCC 1523, require information about detection and morphology.

Furthermore, *Papiliomyceslongiclavatus* is similar to *Papiliomycesshibinensis* in that it has clavate stromata with cylindrical stipe, immersed perithecia and filiform ascospores. However, it differs from *Papiliomycesshibinensis* in that it produces longer ascospores, cylindrical secondary ascospores, two types of phialides and two types of conidia (Table 2). It differs from *Paecilomycesverticillatus* in that it produces longer phialides, longer α-conidia and longer β-conidia. In the phylogenetic tree, *Papiliomyceslongiclavatus* is distinct from *Papiliomycesshibinensis* and *Paecilomycesverticillatus*, which cluster in different clades (Fig. 3). Thus, *Papiliomyceslongiclavatus* is a distinct *Papiliomycesspecies* (Table 2).

## Supplementary Material

XML Treatment for Papiliomyces longiclavatus

## Figures and Tables

**Table 1.** List of taxa and their GenBank accession numbers used in this study.

Species	Strains	Locality	Substrate	*SSU*	*LSU*	*TEF-1α*	*RPB1*	*RPB2*	*ITS*	References
*Keithomycescarneus*	CBS 399.59	USA	Soil	EF468989	EF468842	EF468788	EF468895	EF468939	MT078887	Spatafora et al. (2007)
*Keithomycescarneus*	CBS 239.32	France	Sand dune	EF468988	EF468843	EF468789	EF468894	EF468938	AY624171	Spatafora et al. (2007)
*Paecilomyces* sp.	HR1-11	South Korea	Cymbidium kanran						KU141150	Hong,J.W et al. (2015)
*Paecilomyces* sp.	SC0924	China	Soil						KR011745	Xu et al. (2015)
*Paecilomycesverticillatus*									DQ836182	Han et al. (2006)
*Papiliomycesliangshanensis*	EFCC 1523	Korea	Lepidoptera	EF468961	EF468814	EF468755	–	EF468918	–	Sung et al. (2007)
*Papiliomycesliangshanensis*	EFCC 1452	Korea	Lepidoptera	EF468962	EF468815	EF468756	–	–	–	Sung et al. (2007)
*Papiliomyceslongiclavatus*	GZUH YC20061403	China	Lepidopteran larva	MZ702112	MZ702101	MZ955880	MZ955876	OM419142	MZ702080	This study
*Papiliomyceslongiclavatus*	HKAS 115914	China	Lepidoptera larva	MZ702114	MZ702103	MZ955882	MZ955878	OM419143	MZ702082	This study
*Papiliomycesshibinensis*	GZUH SB13050311	China	Lepidoptera	KR153588	–	KR153589	KR153590	–	–	Wen et al. (2015)

**Table 2.** Synopsis of *Papiliomyces* species discussed in the paper.

Species	*Papiliomycesshibinensis*	*Paecilomycesverticillatus*	*Papiliomyceslongiclavatus*
Stromata	Stromata 42 mm long, 2–3 mm wide, growing from the head of Lepidoptera larva, simple.		Stromata arising from the head of host, ovary, clavate, solitary, 40–60 mm long, 3–5 mm thick.
Stipe	Stipe 17–20 mm long, 2–3 mm wide, flexuous, white to faint yellow.		Stipe cylindrical, greyish-white to light yellow, fleshy, glabrous, enlarging abruptly at fertile portion.
Fertile part	Fertile part on the upper 50% of the stromata, 18–22 mm long, 2–3 mm wide, cylindrical or obtuse, faint yellow, differentiated from stipe, without a sterile apex.		Fertile head length 15–21 mm, 4–6 mm thick, grey white to grey black, mature with a clear boundary with the stalk, no sterile tip.
Perithecia	Ascomata crowded, completely immersed, ampulliform, ovoid to oblong, 630–830 × 240–340 μm, curved, with the ostioles opening on the surface of the fertile head.		Ascomata, bottle-shaped, buried, 320–580×110–230 μm
Asci	Asci 130–200 × 5.1–8.3 μm, 8–spored, hyaline, cylindrical, with a prominent apical cap; apical cap 4.7–5.9 × 2.8–3.5 μm		Asci, narrowly cylindrical, 8-spored, hyaline, possessing a prominent apical cap, 140–230×4.8–6.5 μm
Ascospores	Ascospores 120–170 × 1.4–2.1 μm, hyaline, filiform, smooth–walled, multiseptated with cells 4–5.6 μm long.		Ascospores are nearly isometric, transparent and colourless, slender, filiform, smooth, mature and break into secondary ascospores.
Part–spores	Not breaking into secondary ascospores.		Secondary ascospores, 5.2–9.3 × 1.1–1.5 μm
Condiophores	Conidiophores short, hyaline, smooth, up to 60 µm long, mostly arising from aerial hyphae.	Conidiophores hyaline, 9.6–19.8 µm. Phialides 7.8–14.4 × 1.2–2.4 µm, awl–shaped or consisting of a cylindrical basal portion and a thin neck	Two types of phalides: α–phalides and β–phalides. α–phialides 12.6–23.8 × 1.4–2.4 μm, hyaline, smooth, solitary, mostly arising from aerial hyphae, shorter and gradually thinner upwards. β–phalides 28.2–44.5 × 1.2–1.8 μm hyaline, smooth, slender, conical, mostly solitary.
Conidia	Conidia ellipsoidal, ovoid or fusiform, 1–celled, 3.5–5 × 2–3 µm, in long divergent, dry chains.	Conidia hyaline, mostly subglobose to ellipsoidal, 1.2–1.8 × 0.6–1.2 µm; few fusiform, 1.8–3.0 × 1.8–2.4 µm, forming divergent, dry chains or aggregating spore group	α–conidia.1–5.1×1.2–2.5 μm, round, single celled, smooth, colourless and transparent. β–conidia 5.8–9.9×1.3–2.7 μm, fusiform, with sharp ends, single cell and smooth wall.
Reference	Wen et al. (2015)	Li et al. (2006)	This study
